# Dendrimer-based contrast agents for PET imaging

**DOI:** 10.1080/10717544.2017.1399299

**Published:** 2017-11-10

**Authors:** Lingzhou Zhao, Xiangyang Shi, Jinhua Zhao

**Affiliations:** a Department of Nuclear Medicine, Shanghai General Hospital, Shanghai Jiao Tong University School of Medicine, Shanghai, People’s Republic of China;; b College of Chemistry, Chemical Engineering and Biotechnology, Donghua University, Shanghai, People’s Republic of China;; c CQM-Centro de Química da Madeira, Universidade da Madeira, Funchal, Portugal

**Keywords:** Dendrimers, contrast agent, PET, radiolabeling, positron isotope

## Abstract

Positron emission tomography (PET) imaging offers physiological and biological information through the *in vivo* distribution of PET agents for disease diagnosis, therapy monitoring and prognosis evaluation. Due to the unique structural characteristics allowing for facile modification of targeting ligands and radionuclides, dendrimers can be served as a versatile scaffold to build up various PET imaging agents, and significant breakthroughs have been made in this field over the past decades. This review focuses on the recent advances in dendrimer-based contrast agents for PET imaging of cancer, cardiovascular and other diseases. In particular, radiolabeling strategies for different PET isotopes are described in detail. Several challenges involved in clinical translation of radiolabeled dendrimers are also discussed.

## Introduction

Molecular imaging can be described as *in vivo* real-time visualization, characterization and measurement of biological processes at the molecular and cellular levels (Weissleder, 2006; Chen & Chen, 2010; Bao et al., 2013). As a remarkable progress in medical imaging, various molecular imaging modalities have been developed, including magnetic resonance (MR) imaging (Cai et al., 2013; Li et al., 2013, 2014, 2016; Huang et al., 2015), optical imaging (Shen et al., 2013; Phillips et al., 2014; Etrych et al., 2016; van Brussel et al., 2016), positron emission tomography (PET) (Xiao et al., 2012; Xing et al., 2014; Chakravarty et al., 2015; Dimitrakopoulou-Strauss, 2015; Maurer et al., 2016; Lau et al., 2017) and single photon emission computed tomography (SPECT) (Gomes et al., 2011; Bailey & Willowson, 2013; de Smet et al., 2013; Li et al., 2016). Different from traditional imaging to visualize the ultimate states of a disease, molecular imaging is expected to detect abnormity with more precision in an early stage, in which the required molecular imaging agents play a key role (Hellebust & Richards-Kortum, 2012; Huang & Tsourkas, 2013; Gnanasegaran & Ballinger, 2014; Qiao & Shi, 2015). According to the detected signals of molecular imaging agents, particular targets or pathways can be imaged.

The imaging signals can be produced from metal oxides, fluorescent molecules or radionuclides to meet the requirements of different imaging modalities (James & Gambhir, 2012; Kunjachan et al., 2015; Qiao & Shi, 2015). Among these, molecular imaging agents labeled with positron-emitting radionuclides offer an opportunity to noninvasively monitor their biodistribution and pharmacokinetics *in vivo* by PET. Due to its advantage of high sensitivity and quantitative analysis, PET has gained a wide acceptance as a powerful clinical tool in disease diagnosis, prognosis evaluation and therapy monitoring during the last decade (Tomasi & Rosso, 2012; Groheux et al., 2013; Kikuchi et al., 2013; Nogami et al., 2014; Schüle et al., 2016). Furthermore, the intrinsic weakness of PET imaging is the relatively poor spatial resolution (Drzezga et al., 2012; Luehmann et al., 2016), which can be compensated by other imaging modalities with high anatomical resolution. The formed hybrid imaging techniques, such as PET/CT and PET/MR imaging, strongly drive the development of molecular imaging in more aspects (Hillner et al., 2015; Ohno et al., 2015; Botsikas et al., 2016). However, few new molecular imaging agents, especially multifunctional contrast agents for hybrid imaging, have been approved for clinical applications during the last decade (Hall et al., 2010; Lahooti et al., 2016). Therefore, to meet the growing demands from molecular imaging with improved imaging quality and detection specificity, it is essential to exploit novel PET imaging agents for this powerful technique.

The continuous advances in nanotechnology have shown that various nanoparticles (NPs) can be exploited as PET imaging agents, including but not limited to liposomes (Silindir et al., 2012; Abou et al., 2013; Emmetiere et al., 2013; Rokka et al., 2016; Malinge et al., 2017), micelles (Xiao et al., 2012; Starmans et al., 2015), polymers (Allmeroth et al., 2013; Lee et al., 2013; Luk & Zhang, 2014; Elsabahy et al., 2015; Ma et al., 2016), gold NPs (Xiao et al., 2012; Karmani et al., 2014), metal oxide NPs (Penelope et al., 2012; Pellico et al., 2016; Sun et al., 2016) and dendrimers (Ghobril et al., 2012; Ren et al., 2016; Pant et al., 2017; Smith & Gambhir, 2017). Several radiolabeled nanoparticles have entered preclinical and clinical settings (Stockhofe et al., 2014; Choi et al., 2016). Among these developed nanomaterials, dendrimers (Lee et al., 2005), a class of highly branched, monodispersed, synthetic macromolecules with well-defined architecture and composition and highly controllable size and surface properties have attracted a great deal of attention. Compared to other type of nanostructures, dendrimers offer several key advantages: (1) precise molecular structures and exact number of terminal groups; (2) multiple sites of attachment for various convenient surface modifications; (3) excellent stability and small size with favorable biocompatibility, for example, the size of the most studied generation five dendrimers is only 5.4 nm which enables the direct elimination by renal system without degradation *in vivo*. These unique features enable dendrimers to be conveniently used for construction of nanoscale contrast agents (Lee et al., 2005; Mintzer & Grinstaff, 2011; Peng et al., 2012; Wen et al., 2013; Zhao et al., 2015; Luo et al., 2016), in particular nanoprobes using different positron-emitting nuclides. Furthermore, the physical size and structure of dendrimers are frequently utilized to adjust their *in vivo* excretion behavior and circulation time and to obtain a suitable visualization of passive targeting behavior through enhanced permeability and retention (EPR) effect in specific areas (Kobayashi et al., 2003; Lei et al., 2008; Tang et al., 2013), like tumors. Another approach to increase targeting efficiency in tumors is to build dendrimer-based NPs functionalized with multiple targeting ligands (Liu et al., 2013; Sunoqrot et al., 2014; He et al., 2015). So these ligand-modified dendrimers can have higher probability to target specific receptors over-expressed in tumor cells. Besides, through appropriate surface modification, dendrimers are able to obtain desired water solubility and biocompatibility (Shi et al., 2007; Cheng et al., 2011), which may impart the dendrimer-based PET imaging agents a wide range of applications in clinical practice.

The unique characteristics of dendrimers allow the generation of many PET imaging agents. It has to be emphasized that several key issues have to be considered, such as appropriate isotopes, efficient radiolabeling strategies and beneficial pharmacokinetic profiles, in order to achieve expected objectives. In this review, we describe various radiolabeling strategies for different PET isotopes, and summarize recent advances in the development of dendrimer-based contrast agents for PET imaging. Some challenges and future outlooks related to this area of research are also briefly discussed.

## PET isotopes

Up to date, a great variety of positron-emitting isotopes become available for PET imaging (Decristoforo, 2012; Jødal et al., 2014). Generally, they can be produced by medical cyclotrons or obtained from specific generators and classified into two categories according to their physical half-lives. Short-lived positron emitters include ^15 ^O, ^13 ^N, ^11 ^C, ^18 ^F and ^68^Ga with half-lives from 2 min to 110 min (Mirshojaei et al., 2016), which is compatible for measurements within an initial time frame. Long-lived positron emitters with half-lives of several hours or days (Stockhofe et al., 2014), such as ^64^Cu, ^76^Br, ^89^Zr, ^124^I and ^74 ^As, can be applicable for relatively slow processes and certain effects, like EPR effect. The typical radionuclides for PET imaging and their common production methods are summarized in Table 1.

**Table 1.** Representative radioisotopes for PET imaging and their production methods.

Radioisotopes	Half-life	β^+^ properties (%)	β^+^ *E*_max_ (MeV)	Productionmethods
^11^C	20.4 min	99.8	0.96	Cyclotron
^13^N	10.0 min	99.8	1.199	Cyclotron
^15^O	2.0 min	99.9	1.732	Cyclotron
^18^F	109.8 min	97.0	0.635	Cyclotron
^64^Cu	12.7 h	17.8	0.653	Cyclotron
^68^Ga	67.8 min	89	1.899	Generator
^76^Br	16.2 h	55	3.94	Cyclotron
^89^Zr	78.4 h	22.7	1.81	Cyclotron
^124^I	4.2 d	23	0.91	Cyclotron
^74^As	17.8 d	29	1.54	Cyclotron

Considering the crucial role of physical half-life in PET imaging, appropriate PET isotopes and their efficient radiolabeling strategies must be carefully taken into consideration in order to obtain optimal results (Sun et al., 2007; Stockhofe et al., 2014). Distinctly different from the normal chemical reaction, the radiolabeling synthesis is usually time-constrained; therefore, fast reaction time and simple procedure for purification are indispensable, especially for those short half-life isotopes. At the same time, only a trace amount of radioactive isotopes is added to label with excess precursors; on one hand, the synthesis can benefit from the abundant precursors to increase the radiolabeling yield; on the other hand, the radiolabeling reaction becomes very sensitive and prone to be slowed down or even stopped by a tiny amount of impurities or other reactive functional groups in precursors. In general, as to the very short-lived isotopes ^15 ^O (*t*_1/2_=2.0 min) and ^13 ^N (*t*_1/2_=10.0 min), they are mainly used in simple forms such as ^15^O_2_, H_2_^15^O, C^15^O_2_ or ^13^NH_3_, to determine blood flow and volume distribution (Danad et al., 2013; Hori et al., 2014; Kim et al., 2015). In comparison to ^15 ^O and ^13 ^N, ^11 ^C (*t*_1/2_=20.4 min) has a relatively longer half-life with a wide range of applications. ^11 ^C can be introduced into an organic structure by methylation which is often achieved using ^11 ^C-iodomethane. By replacing a nonradioactive ^12 ^C atom, ^11 ^C can be easily labeled with biological molecules, including but not limited to amino acids, nucleic acids, choline and dopamine (Okada et al., 2011; Villemagne et al., 2012; Umbehr et al., 2013; D'Souza et al., 2014). Thanks to the same structure before and after labeling, the chemical and biological properties of biological molecules are not changed, which can faithfully reflect their distribution, metabolism and excretion in the body. Nevertheless, due to the limitation of their short half-lives, only a very few of ^13 ^N- and ^11 ^C-labeled NPs have been reported (Pérez-Campaña et al., 2013; Sharma et al., 2013).

Currently, ^18 ^F is the most commonly used PET isotope with a modest half-life (109.8 min), while the addition of ^18 ^F to complex molecules is still challenging. Usually, the ^18 ^F radiolabeling is carried out through the nucleophilic substitution of some excellent leaving groups in precursors such as mesylate, tosylate or triflate. Despite many ^18 ^F-labeling methods have been proposed, most of them suffer from harsh reaction conditions, multistep protocols and low radiochemical yields, which is also a major obstacle of ^18 ^F-labeled NPs (Devaraj et al., 2009; Liu & Welch, 2012). For covalent binding of ^18 ^F onto NPs, either direct or prosthetic group radiolabeling is often impossible or provides only poor overall yields. Consequently, alternative labeling strategies have been developed for fast, stable and high yielding of radiosynthesis. Taking click chemistry as an example (Zeng et al., 2013; Meyer et al., 2016), through the copper-catalyzed azide-alkyne cycloaddition reaction, ^18 ^F can be efficiently and mildly conjugated to azide-modified NPs. Radiohalogens, such as ^76^Br and ^124^I, have half-lives of 16.2 h and 4.2 days, respectively, which allows a prolonged time frame for scanning. In contrast to ^18 ^F, radiolabeling of NPs with ^76^Br or ^124^I can be convenient and efficient *via* Chloramine T method (Sundin et al., 1999; Taldone et al., 2016), for instance, by the introduction of tyrosine into NPs. It is well known that the first-in-human clinical study of radiolabeled nanoparticles for cancer imaging was done with ^124^I-labeled ultrasmall inorganic hybrid nanoparticles (Phillips et al., 2014).

It is worth noting that ^64^Cu and ^68^Ga have been the most extensively researched radiometals in the construction of radiolabeled NPs because of latent chemical properties, favorable decay half-life, and commercial availability (Zeng et al., 2012; Banerjee et al., 2014). Conveniently, ^68^Ga and ^64^Cu can be conjugated on the surface of NPs through coordination chemistry. Since NPs do not have any metal binding sites, bifunctional chelators (BFCs) are indispensable, which forms stable complexes with these radiometals. As the well-established coordination chemistry, a wide range of BFCs have been designed and synthesized, which greatly facilitates the development of various radiometal-labeled NPs for PET-imaging applications (Wadas et al., 2010). 1, 4, 7, 10-Tetraazacyclododecane-1, 4, 7, 10-tetraacetic acid (DOTA) as a typical representative of macrocyclic chelating agents, a widely used chelator, has been often connected with dendrimers to chelate nonradioactive Gd(III) for MR imaging (Chen et al., 2015; Mustafa et al., 2016). This chelating system allows simultaneous coupling of different types of imaging elements in one NPs for multimodal imaging applications (e.g. PET/MR) (Park et al., 2010; Pellico et al., 2016). Furthermore, *via* substituting the diagnostic radionuclide with a therapeutic one, whereas the chelator and the nanodimensional structure remain, it is possible to build various theranostic nanoplatforms (Weineisen et al., 2015; Petersen et al., 2016).

## Dendrimer-based PET imaging agents

### ^18^f

^18 ^F is regarded as an ideal positron emitter for PET imaging, due to its high positron intensity (97%) and optimal positron emission energy (0.635 MeV). This means a short positron linear range in tissue that enables high-resolution PET imaging (Conti & Eriksson, 2016). Besides, the proper half-life (109.8 min) is favorable for imaging quality and radiation safety. Thanks to these benefits, abundant ^18 ^F-labeled agents have been developed for different clinical applications in the past decades; however, ^18 ^F is mostly used in the form of ^18 ^F-fluorodeoxyglucose (^18 ^F-FDG) for tumor imaging (Hall et al., 2010; Kurihara et al., 2012). In the meantime, with the popularization of PET imaging and daily production through cyclotrons in most major hospitals, ^18 ^F becomes the most readily available PET isotope, which greatly boosts the research of ^18 ^F-radiolabeled NPs (Stockhofe et al., 2014; Xing et al., 2014).

Trembleau et al. first showed that dendrimers could be labeled with ^18 ^F-fluorinatable groups at room temperature (Trembleau et al., 2011). The dendrimers used in this study possessed a disulfide linker, which could subsequently generate two dendrons with thiol groups for conjugation with biotin. To facilitate ^18 ^F-fluorination at room temperature in aqueous solvent, trifluoro-boroaryl moieties were attached to the terminal NH_2_ groups of dendrimers. After incubation of the boroaryl functionalized dendrons with ^18 ^F-fluoride in glacial acetic acid, the radiochemical yield could be up to 55%. This rate was similar for dendrons with 4, 8 and 16 branches. These ^18 ^F-dendron-biotins displayed targeting specificities to HER-2 expressing cells pretargeted with avidin-trastuzumab *in vitro*. Unexpectedly, the cell-associated activity of ^18 ^F-dendron-biotin decreased significantly with increasing dendron size, suggesting that larger dendrons might sterically hinder binding of avidin with biotin. Although the developed radiolabeling method might be suitable for the temperature-sensitive nanomaterials, there remains challenges to seek appropriate linkers for the conjugation of targeting molecules to improve specificity.

### ^76^Br

**Figure 1.**
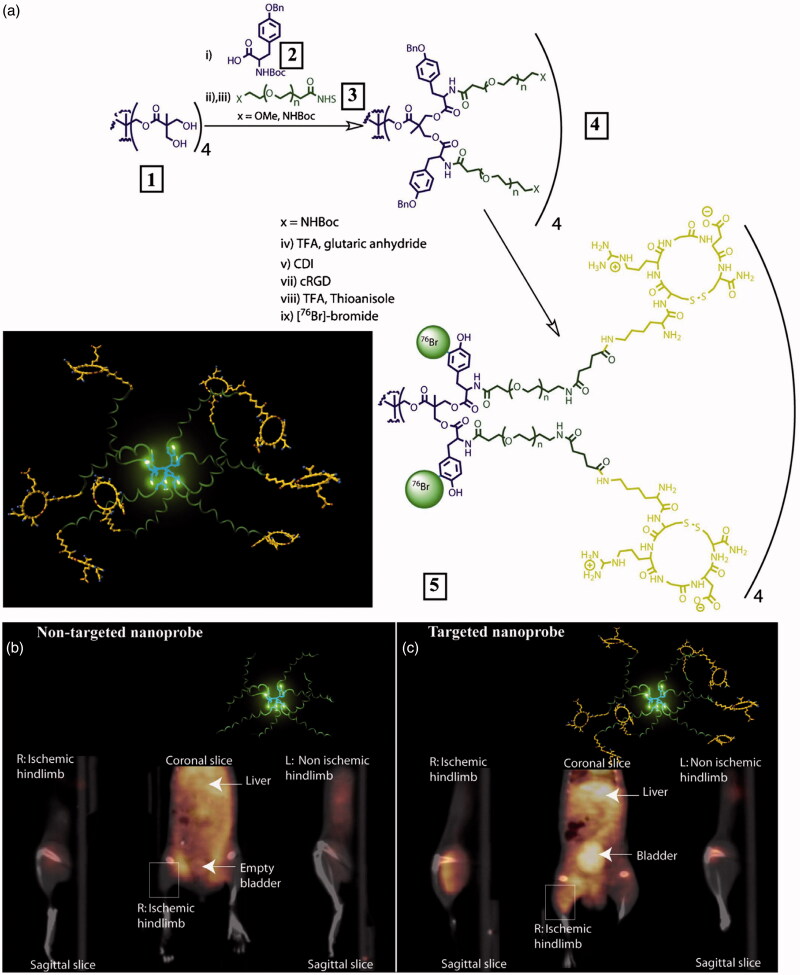
(a) Preparation of PET nanoprobes targeted at α_v_β_3_ integrin. (b) Noninvasive PET/CT images of angiogenesis induced by hindlimb ischemia in amurine model for nontargeted dendritic nanoprobes (shown bottom center). (c) Noninvasive PET/CT images of angiogenesis induced by hindlimb ischemia in a murine model for α_v_β_3_-targeted dendritic nanoprobes, which showed higher uptake in ischemic hindlimb (left side of image) as compared with control hindlimb (right side of image) (adapted from Almutairi et al., 2009).

^76^Br is known to be a positron emitter with 57% positron emission and 43% electron capture with relatively long half-life (16.2 h), which has been used to label antibody and small molecules for PET imaging (Jagoda et al., 2012; Hanaoka et al., 2015). ^76^Br can be labeled with high radiolabeling yield at room temperature in a short time. Almutairi et al. reported a unique approach to build up ^76^Br-labeled biodegradable dendrimers for PET imaging of angiogenesis (Figure 1) (Almutairi et al., 2009). The developed nanoprobe was manufactured using pentaerythritol as the dendritic core to functionalize with tyrosine groups for ^77^Br labeling and heterobifunctional polyethylene oxide chains (PEO) forming protective shells to prevent dehalogenation *in vivo*. Radiolabeling was achieved using Chloramine-T method and the ^76^Br-labeled dendrimers displayed excellent stability in PBS and mouse serum within 48 h. Moreover, the pharmacokinetics could be modulated through appropriate level of dendritic branching and PEO length. RGD peptides were modified with lysine and could be further installed at the ends of the PEO chains. The targeted nanoprobes exhibited a 50-fold enhancement in binding affinity over the mono-RGD peptide and 6-fold increase in α_v_β_3_ receptor-mediated endocytosis compared with nontargeted nanoprobe. Highly specific accumulation of ^76^Br-labeled dendritic nanoprobe was found in a murine hindlimb ischemia model and the potential of dendritic nanoprobe as a PET-imaging agent of angiogenesis was verified *in vivo*. Remarkably, the design of protective shells in this study could be potentially used to improve radiostability of radioiodine-labeled nanoprobes that are more vulnerable to dehalogenation *in vivo*.

### ^64^Cu

^64^Cu can be conceptually used for both imaging and potential therapy in nuclear medicine field, due to its specific nuclear properties, such as a favorable half-life (12.7 h) and attractive decay characteristics (β^+^, 17.8%; β^−^, 38.4%; EC, 43.8%) (Conti & Eriksson, 2016). As a radiometal, ^64^Cu requires a BFC for attaching it to NPs. Apart from DOTA, 1,4,7-triazacyclononane-1,4,7-triacetic acid (NOTA) and 1,4,8,11-tetraazacyclotetradecane-N,N′,N″,N″″-tetraacetic acid (TETA) are additional macrocyclic chelators (Stockhofe et al., 2014). Normally, the radiolabeling protocols share similar reaction conditions and purification methods. Through a ^64^Ni(p,n)^64^Cu nuclear reaction in cyclotron accelerator, ^64^CuCl_2_ is provided in a hydrochloric acid solution, and then mixed with the NPs dissolved in nearly neutral or weakly acidic buffer solution. The mixture is incubated at room temperature or heated to higher temperatures for one half to several hours. The crude products can be purified by adding another chelating agent (e.g. DTPA), gel filtration chromatography (e.g. PD-10 desalting column) or centrifuged filtration to remove free ^64^Cu.

**Figure 2.**
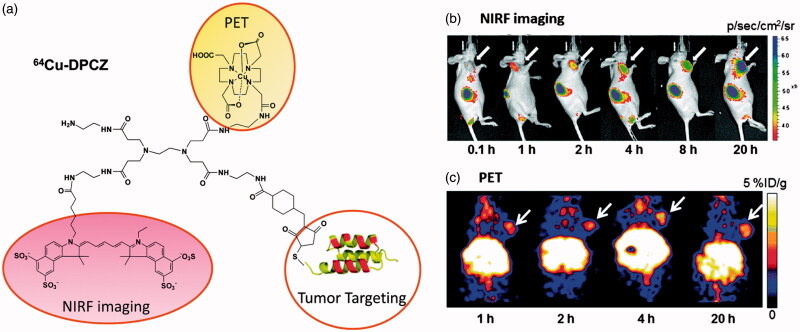
(a) Schematic structure of ^64^Cu-DPCZ which is constituted by four components, PAMAM G0 as a scaffold, Cy5.5 as an optical reporter, ^64^Cu-DOTA as a PET reporter and Affibody as a tumor-targeting molecule; (b) *In vivo* NIRF imaging of SKOV3 tumor-bearing mice at 0.1, 1, 2, 4, 8 and 20 h after tail vein injection of ^64^Cu-DPCZ; (c) Decay-corrected coronal micro-PET images of mice bearing SKOV3 tumor at 1, 2, 4 and 20 h after tail vein injection of ^64^Cu-DPCZ. Arrows indicate the location of the tumors (*n* = 3) (adapted from Wang et al., 2014).

Wang et al. developed an anti-HER_2_ Affibody-based dual imaging probe using PAMAM generation 0 (PAMAM G0) as a platform to assemble ^64^Cu and Cy5.5 for dual-modality imaging of ovarian cancer (Figure 2) (Wang et al., 2014). The PAMAM G0 molecule contained four peripheral amines and could be readily coupled with Cy5.5-NHS and DOTA-NHS *via* the formation of acylamide. The anti-HER_2_ Affibody was then connected with PAMAM through a bifunctional linker, sulfosuccinimidyl-4-(N-maleimidomethyl) cyclohexane-1-carboxylate (Sulfo-SMCC), which could react with the amine terminal groups of G0 and coupled with the cysteine in anti-HER_2_ Affibody. The developed dendrimer conjugates could effectively chelate with ^64^Cu, but the ^64^Cu-labeled complexes were not very stable *in vivo*. The main reason of instability is considered to be the reduction of Cu(II) to Cu(I) in the ^64^Cu-DOTA moiety, while DOTA is unsuitable for the chelation of Cu(I), generating transchelation between ^64^Cu-DOTA and some proteins such as serum albumin and superoxide dismutase. Nevertheless, both NIRF and PET imaging exhibited high tumor uptake with obvious contrast effects at 1 h post injection, and excellent tumor imaging results were observed within 20 h, which attributed to favorable pharmacokinetic properties. Interestingly, tumor fluorescence signals gradually increased during the period investigated, whereas a radioactivity peak from PET were found at 4 h after injection. This difference might be explained by the fact that NIRF and PET followed the fate of Cy5.5 and ^64^Cu moieties, respectively. Biodistribution studies showed that this dendrimer-based dual-modality imaging probe is accumulated prominently in liver and kidneys, suggesting the excretion through both hepatobiliary and kidney systems.

**Figure 3.**
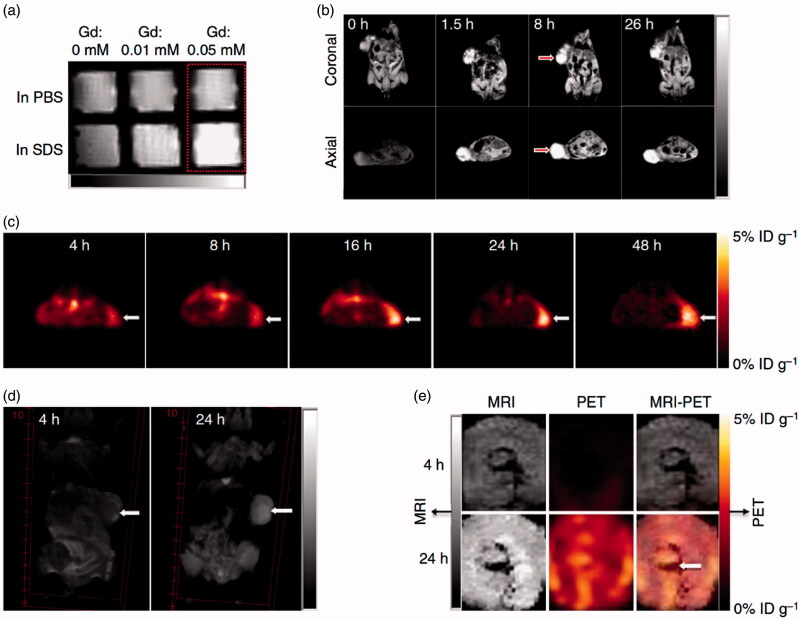
Nanoporphyrin-mediated MRI and PET imaging in animal models. (a) *In vitro* MRI signal of Gd-NPs in the absence and in the presence of SDS obtained by T1-weighted MRI on a Bruker Biospec 7 T MRI scanner using a FLASH sequence. (b) Representative coronal and axial MR images of transgenic mice with mammary cancer (FVB/n Tg(MMTV-PyVmT) using a FLASH sequence preinjection and after injection of 0.15 ml Gd-NPs (Gd dose: 0.015 mmolkg^−1^). The white arrow points to the tumor site. (c) PET image of nude mice bearing SKOV3 ovarian cancer xenografts at 4-, 8-, 16-, 24- and 48-h post-injection of ^64^Cu-labeled NPs (150–200 μl, ^64^Cu dose: 0.6–0.8 mCi). The white arrow points to the tumor site. (d) 3 D coronal MR images of nude mice bearing A549 lung cancer xenografts using a FLASH sequence at 4- or 24-h post-injection with 0.15 ml of 64Cu and Gd dual-labeled NPs (150–200 ml, ^64^Cu dose: 0.6–0.8 mCi, Gd dose: 0.015 mmol kg^−1^). The white arrow points to the tumor site. (e) PET-MR images of tumor slices of nude mice bearing A549 lung cancer xenograft at 4- or 24-h post-injection of dual-labeled NPs. White arrow points to the necrotic area in the center of the tumor (adapted from Li et al., 2014).

In another study, Li et al. developed smart and versatile telodendrimers consisting of various imaging and therapeutic functions such as NIRF, PET and MR imaging, photothermal therapy (PTT), photodynamic therapy (PDT), as well as image-guided drug delivery (Li et al., 2014). This multifunctional nanoplatform was synthesized by the self-assembly of hybrid amphiphilic polymers comprising linear polyethylene glycol (PEG), dendritic oligomers of pyropheophorbide-a (a porphyrin analog, Por) and cholic acid (CA). In order to improve the structural stability of NPs in blood circulation, four cysteines were introduced to the oligolysine backbone of the telodendrimers and then crosslinked *via* disulfide bond. Due to the structure feature of porphyrin components, the self-assembled telodendrimers possessed an intrinsic ability to chelate ^64^Cu for PET imaging or Gd(III) for MR imaging (Figure 3). Notably, the radiolabeling strategy was very simple and fast. Only after incubation of telodendrimers with ^64^CuCl_2_ solution for 30 min at room temperature, the radiochemical yields could be up to 96.5%. When excited at 405 nm, the telodendrimers displayed a weak red-fluorescence emission at 680 nm, but very strong fluorescence in the presence of sodium dodecyl sulfate (SDS). Similar to the fluorescence property, the telodendrimers also possessed the ability of photodynamic transduction. After laser irradiation, telodendrimers could convert energy in the form of heat in PBS, while fluorescence and singlet oxygen generation with the addition of SDS. Furthermore, chemotherapeutic drugs could be efficiently encapsulated inside the telodendrimers as programmable releasing nanocarriers for drug delivery. These properties enabled telodendrimers as theranostic agents for NIRF imaging, PTT, PDT and chemotherapy, which had been demonstrated in both ovarian cancer xenograft model and murine transgenic breast cancer model *in vivo*.

**Figure 4.**
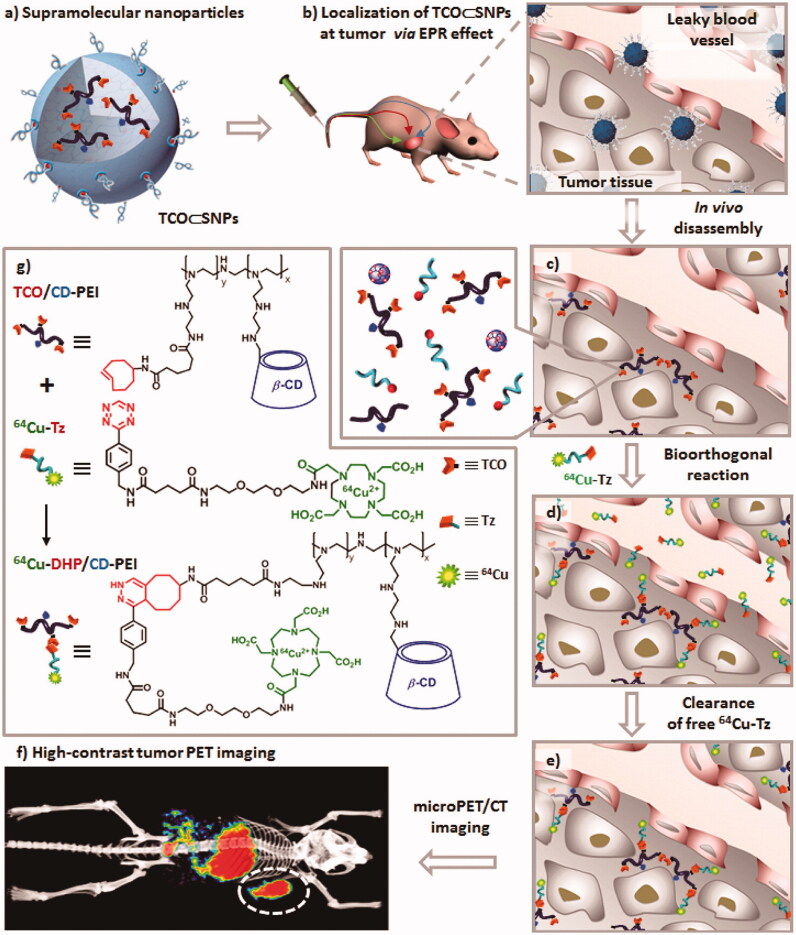
Schematic representation of a new approach for pretargeted PET imaging that leverages the utilities of supramolecular nanoparticles (SNPs) and bioorthogonal chemistry: (a) Supramolecular synthetic strategy is employed for preparing the tumor-targeting agent (TCO⊂SNPs); (b) after intravenous injection, the tumor EPR effect drives preferential accumulation of TCO⊂SNPs in tumor; (c) after TCO⊂SNPs have accumulated in tumor, TCO⊂SNPs disassemble to release a TCO-grafted molecular building block, TCO/CD-PEI; (d) a radiolabeled reporter (^64^Cu-Tz) is then injected for bioorthogonal reaction with tumor-retained TCO/CD-PEI; (e) the unreacted ^64^Cu-Tz was cleared quickly from the body; (f) the resulting dihydropyrazine (DHP) conjugation adduct (^64^Cu-DHP/CD-PEI) confines radioactivity in tumor, resulting in high-contrast tumor PET imaging. (g) Chemical structures of the bioorthogonal reactions between TCO/CD-PEI and ^64^Cu-Tz (adapted from Hou et al., 2016).

The development of PET nanoprobes is vulnerable to be restricted by the contradiction between intrinsic pharmacokinetics (PKs) of NPs and limited half-lives of positron-emitting isotopes. To this issue, the pretargeted imaging strategy may be one of the promising solutions (Zeglis et al., 2013; van Duijnhoven et al., 2015; Hou et al., 2016). In an ideal pretargeted PET-imaging system, the tumor-targeting agents should preferentially accumulate in tumors within a reasonable time frame. Then, radiolabeled ligands can effectively distribute to whole body and irreversibly combine the tumor-targeting agents previously accumulated in tumor sites. In parallel, uncombined radioligands were cleared rapidly throughout the body, as revealed by high-contrast tumor PET imaging. In a recent study, Hou et al. reported tumor-targeting supramolecular NPs (TCO⊂SNPs) for pretargeted PET imaging (Figure 4) (Hou et al., 2016). TCO⊂SNPs were self-assembled by cyclodextrin-polyethylenimine polymer (CD-PEI), *trans*-cyclooctene modified CD-PEI (TCO/CD-PEI), adamantane-grafted polyamidoamine (Ad-PAMAM) and Ad-grafted polyethylene glycol (Ad-PEG). The ^64^Cu labeling of TCO⊂SNPs was designed *via* Diels-Alder reaction between TCO and tetrazine-DOTA-^64^Cu (^64^Cu-Tz). ^64^Cu-Tz could be prepared with high radiochemical yield and its stability was up to 95% with 8 h both in PBS and serum. TCO groups were encapsulated into supramolecular NPs to be protected from potential degradation *in vivo*. When preferential accumulation of TCO⊂SNPs in tumor through EPR effect occurred, TCO/CD-PEI could be released from the inside of TCO⊂SNPs and reacted with ^64^Cu-Tz to retain radioactivity in tumor. After the quick clearance of the unreacted ^64^Cu-Tz from the body, high-contrast tumor PET imaging were achieved. In contrast to traditional nanoparticle-based imaging platforms with faint tumor uptake and excessive liver distribution, the pretargeted approach showed approximately equivalent uptake in tumor and liver.

Aside from cancer imaging, PET imaging of cardiovascular and inflammatory diseases are gaining importance in the field of molecular imaging (Ratib et al., 2013; Hess et al., 2014; LaForest et al., 2016; Chen et al., 2017). Seo et al. reported a ^64^Cu-labeled dendrimer for PET imaging of atherosclerotic plaque (Seo et al., 2014). They demonstrated that LyP-1, a cyclic 9-amino acid peptide, was able to bind to p32 protein, a biomarker in the progression of atherosclerosis, but the binding affinity of LyP-1 was relatively low in aorta (Hamzah et al., 2011). To improve the accumulation efficacy in atherosclerosis, a dendritic form of LyP-1 was designed and synthesized using lysine as a core structural element. 6-BAT (an analog of TETA) was attached to the dendrimer *via* free thiol groups of C-terminal cysteine for labeling of ^64^Cu, and the decay corrected radiochemical yield was 80 ± 5.7% (*n* =3). The ^64^Cu-labeled dendritic peptide showed significantly enhanced accumulation in atherosclerotic plaque and higher aorta/blood ratio as compared with both the monomer and control peptide through *in vivo* PET imaging. In another study, Pant et al. exploited ^64^Cu-labeled dendritic polyglycerol sulfates (dPGS) as inflammation-specific agents for PET imaging (Pant et al., 2015). It was noted that through facile modification of 1,4-bis(2-pyridinylmethyl)-1,4,7-triazacyclononane (DMPTACN) with isothiocyanate or maleimide groups, two novel types of copper(II)-chelating ligands could directly couple with amino or sulfhydryl groups of dPGS. The formed dPGS-DMPTACN could be effectively labeled with ^64^Cu with a yield of 99% and displayed excellent radiostability *in vitro* within 24 h. However, PET imaging and biodistribution studies of the ^64^Cu-labeled dPGs were only carried out in healthy rats, and further evaluations of these potential inflammation-specific agents in inflammatory models have not been investigated.

### ^68^Ga

As a nonphysiologic metallic positron emitter, ^68^Ga has attracted considerable attention because of the availability from ^68^Ge/^68^Ga generator, low production cost and convenient labeling strategy (Fani et al., 2008). Furthermore, in terms of decay characteristics, ^68^Ga (β^+^, 89%; EC, 11%) shows significant superiority over ^64^Cu (β^+^, 17.8%; β^−^, 38.4%; EC, 43.8%) to gain improved image quality in theory for PET imaging and more suitable half-life of 67.8 min for clinical applications (Conti & Eriksson, 2016). Similar to ^64^Cu, ^68^Ga can be chelated with DOTA and NOTA. Even when the same chelators are used, the ^68^Ga-labeling methods quite varied due to the diverse labeled structures. Recently, Ghai et al. described the optimal radiolabeling of ^68^Ga with PAMAM G4 dendrimer-DOTA conjugate (Ghai et al., 2015). The best radiolabeling efficiency of 96.8% was achieved at pH 4.0, 30 min of incubation time and reaction temperature between 90 and 100 °C. The radiolabeled dendrimers remained stable (with radiolabeling efficiency of 96.0%) for up to 4 h *in vitro* and serum, and the plasma protein binding was observed to be 21.0 ± 3.4%. PET imaging showed that this ^68^Ga-labeled dendrimers could be efficiently retained in tumor tissues through EPR effect and excreted primarily through kidneys.

**Figure 5.**
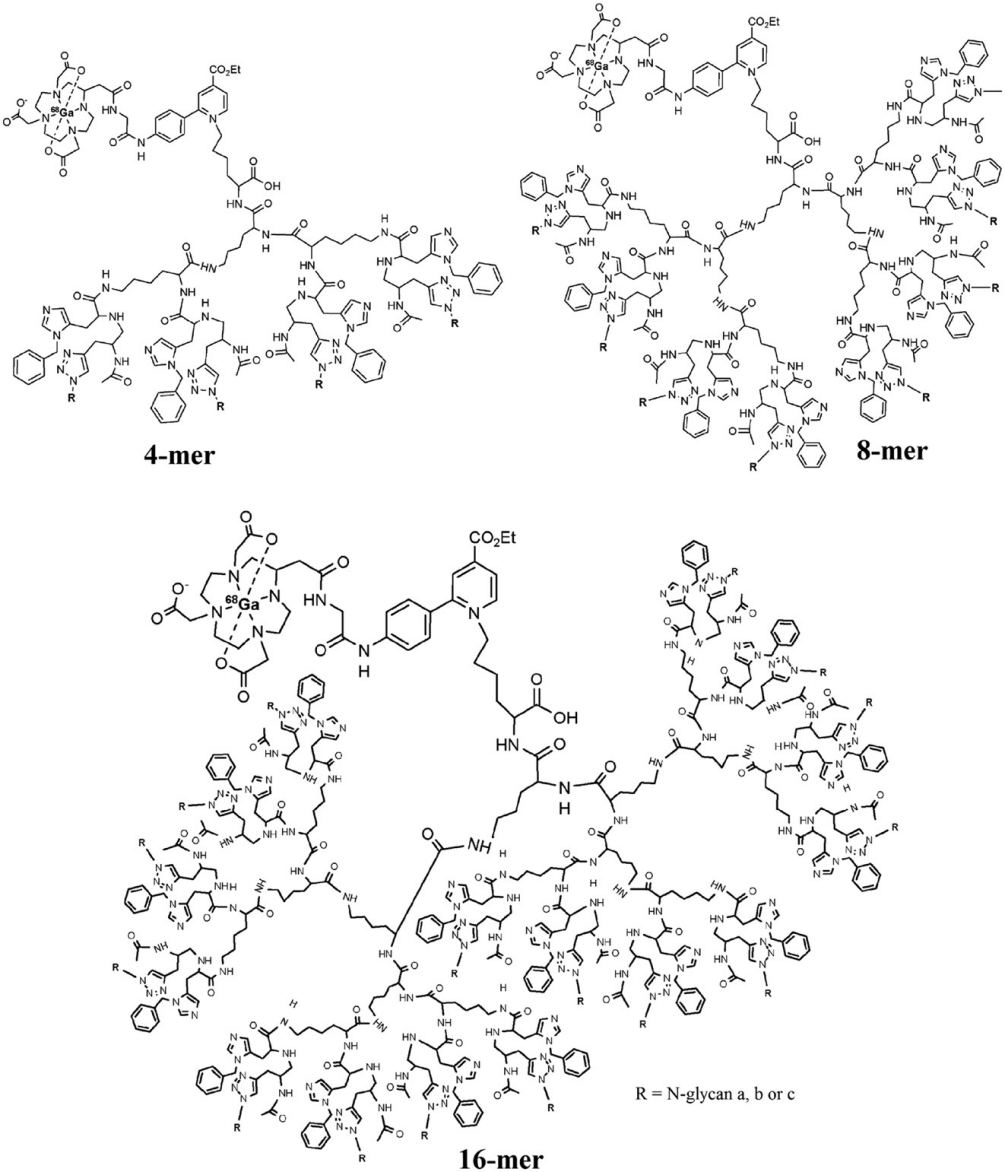
Generation and structure of N-glycan (bis-Neua(2-6)Galglycan (a), bis-Neua(2-6)Gal-asialo glycan (b) and bis-Neua(2-3)Galglycan (c)) clusters labeled with ^68^Ga-DOTA (adapted from van Ghobril et al., 2012).

Tanaka et al. reported PET imaging of dendrimer-type asparagine-linked oligosaccharide (N-glycan) clusters to visualize their dynamics and biodistributions *in vivo* (Tanaka et al., 2010). In this work, different generations of glycoclusters consisting of 4, 8 and 16 molecules of N-glycan derivatives, were prepared (Figure 5). The hexadeca-glycoclusters (16-mers) had three kind of structures due to the composed different N-Glycans, bis-Neua(2-6)Gal-containing glycan (a), asialo glycan (b) and bis-Neua(2-3)Gal-glycan (c), respectively, while bis-Neua(2-6)Gal-containing glycan was only used in tetra-glycocluster (4-mer) and octa-glycocluster (8-mer). DOTA could be linked with terminal amine group of lysine in these glycoclusters for ^68^Ga labeling. The PET results in normal mice showed differences in the clearance properties between the 4-mer, 8-mer and 16-mers, probably due to their molecular size. Smaller glycoclusters of 4-mer and 8-mer could be rapidly and almost completely cleared through kidney, but 16-mer-a was eliminated from urinary bladder and gallbladder with a slow rate. In addition, difference in the biodistribution between 16-mer-b and 16-mer-c were also observed. Unlike the case of 16-mer-a, glycocluster 16-mer-b was cleared through the kidney to the bladder with some accumulation in the liver, and 16-mer-c was rapidly cleared through the kidney/urinary bladder. These results implied that the Neua(2-6)Gal linkage in glycoclusters played an important role in the circulatory residence of N-glycans and varied remarkably in the clearance pathway from those of glycoclusters of 16-mer-b and 16-mer-c, which were cleared through a biofiltration pathway in the kidney.

Radiolabeled arginine–glycine–aspartic acid (RGD) peptide that can target α_v_β_3_ integrin receptors has been widely used in the fields of cancer and cardiovascular diseases (Dijkgraaf et al., 2011; Zhu et al., 2012; Wang et al., 2015; Zhai et al., 2015; Chen et al., 2016; Singh et al., 2016). In comparison with monomers, RGD multimers present an enhanced binding rate and stability *in vivo*, which promotes the development of multimeric RGD peptide radiopharmaceuticals for noninvasive imaging. Several groups have synthesized radiolabeled tetrameric RGD peptides and verified their higher binding affinity and specificity than dimers to tumor cells by PET imaging (Wu et al., 2005, 2007; Dijkgraaf et al., 2011). Then, a further improved integrin-binding affinity with higher initial uptake and longer tumor retention was obtained from a RGD-peptide octamer, which reinforced the theory of the multivalency effect (Li et al., 2007). Nevertheless, the purification of multimers become increasingly difficult with the increase in the number of RGD moieties. To work out the synthetic limits of multimerization, Wängler et al. used PAMAM dendrimers as scaffold to manufacture RGD peptide multimers (Wängler et al., 2010). Three different types of click chemistry reactions were employed to determine the most efficient multimerization approach. Since oxime formation and 1,3-dipolar cycloaddition did not permit the achievement of high multimeric probes, a series of RGD multimers were successfully synthesized by Michael addition of thiols to maleimides, including mono-, di-, tetra-, octa- and, for the first time, hexadecimers. The obtained multimers were conjugated to a DOTA derivative and PEG spacer for accessible ^68^Ga radiolabeling. These cRGD multimers could easily be labeled with ^68^Ga in radiochemical yields between 95% and 98% and radiochemical purities between 96-99%. As expected, binding avidities of RGD multimers constantly was amplified with increasing number of peptide moieties in the *in vitro* studies, as a result, hexadecimers showed a very high avidity to α_v_β_3_ integrin and integrin-expressing U87MG cells, 131 and 124 times higher than in the case of the corresponding monomer, respectively.

This synthesis approach using dendritic structures as scaffolds for multimerization of bioactive molecules was broadened in a following work (Fischer et al., 2014; Lindner et al., 2014). Due to tolerable *in vivo* stability and reasonable tumor uptake, PESIN peptide is regard as one of the most promising ligands to gastrin releasing peptide receptor (GRPR), which is overexpressed on several tumor types. Lindner et al. synthesized a series of PESIN monomers, dimers, tetramers and octamers comprising PEG linkers of different lengths, followed by conjugation with PAMAM dendrimers and efficient radiolabeling of ^68^Ga. The labeled dendrimers could be obtained in high radiochemical yields and purities of 96–99% with excellent stability. The shortest linker within each group (monomers to octamers) resulting in minimal distances between the peptide moieties showed the highest binding affinities *in vitro*. However, the effect of binding affinities increasing proportionally to the number of peptide moieties was not observed in the case of these PESIN multimers. The dimers presented the optimized binding affinity, namely a 2.5-fold avidity enhancement compared to the monomeric peptides, which was further validated by the result that PET study of ^68^Ga-labeled dimers displayed a twice higher tumor uptake in tumor-bearing mice. Interestingly, benefiting from the much faster blood clearance, the dimer also showed twofold high tumor-to-background ratios than the respective monomers.

## Conclusions and outlooks

In summary, dendrimer-based PET imaging agents are reviewed. The typical examples presented in this review demonstrate that positron isotope-labeled dendrimers have a great potential for PET imaging of cancer, cardiovascular and other diseases through targeting ligands (e.g. RGD peptides) or EPR effect. The unique structural characteristics of dendrimers have offered opportunities to incorporate various molecular imaging modalities in a single system for multimodal imaging, such as PET/MR and PET/NIRF imaging. More importantly, therapeutic capabilities are able to be introduced into multifunctional dendrimers as well for theranostic applications. On the other hand, appropriate isotopes and efficient radiolabeling strategies must be carefully considered in the construction of dendrimer-based contrast agents for PET imaging. Interestingly, pretargeted strategy can provide a great deal of flexibility of imaging time, especially for the short-lived isotopes. Several radiolabeling approaches for different PET isotopes have been developed with satisfactory results.

Although positron isotope-labeled dendrimers have shown a great promise in the field of molecular imaging, quite a number of problems need to be solved in their clinical translation (Stylianopoulos & Jain, 2015). The major obstacle is insufficient specificity in target tissues and redundant accumulation in the mononuclear phagocytic system (MPS). One of the promising approaches to increase targeting specificity is to build dendrimer platforms functionalized with monoclonal antibodies, peptides or other targeting ligands that can recognize specific receptors or antigens *in vivo*. In addition, the physical properties of NPs, such as size, shape and surface charge, significantly determine their *in vivo* biodistribution behavior; therefore, dendrimers with optimal surface modifications can prolong circulation time, decrease MPS uptake and have the potential to increase the target-to-background ratio as compared to the uncoated counterparts. Further, the construction of smart and versatile dendrimers for molecular imaging still remains an open area of investigation, for instance, exploring new synthetic techniques specific to dendrimer-based molecular imaging contrast agents. Thus, in order to fulfill PET applications, novel radiolabeling strategies with sufficient radiochemical yields and *in vivo* stabilities must be developed. It should be noted that click chemistry in recent years has shown significant benefits over traditional synthetic methods for the clean, high yielding and rapid preparation of imaging agents labeled with PET radionuclides such as ^18 ^F, ^64^Cu and ^68^Ga. However, the application of click techniques to radiolabeled dendrimer-based NPs is still in an early stage and much more effort is needed in this field. In the aspect of safety, the long-term toxicity of dendrimer-based contrast agents is still confusing, especially the large systems with lack of complete clearance from the body. To resolve this issue, the biodegradable dendrimers will be a better choice. Lastly, due to that fact that dendrimers are able to be loaded with various drugs, genes and therapeutic radionuclides, more types of dendrimer-based theranostic systems could be developed in order to expand the scope of molecular imaging applications, in particular PET image-guided drug delivery (Chakravarty et al., 2014).
